# A Straightforward Procedure for the High-Yield Extraction of Tricin and Flavonoids from Ecuadorian *Huperzia brevifolia* (Lycopodiaceae)

**DOI:** 10.3390/plants14203220

**Published:** 2025-10-20

**Authors:** Chabaco Armijos, Leydy Nathaly Castillo, Jorge Ramírez, Giovanni Vidari

**Affiliations:** 1 Departamento de Química, Facultad de Ciencias Exactas y Naturales, Universidad Técnica Particular de Loja, Loja 1101608, Ecuador; lncastillo@utpl.edu.ec (L.N.C.); jyramirez@utpl.edu.ec (J.R.); 2 Department of Medical Analysis, Faculty of Applied Science, Tishk International University, Erbil 44001, Iraq; vidari@unipv.it

**Keywords:** tricin, flavone, flavonoids, *Huperzia brevifolia*, extraction, HPLC-DAD analysis

## Abstract

Rich natural sources of the flavone tricin (5,7,4′-trihydroxy-3′,5′-dimethoxyflavone; systematic IUPAC name: 5,7-dihydroxy-2-(4-hydroxy-3,5-dimethoxyphenyl)-4*H*-1-benzopyran-4-one) are actively sought since this compound is endowed with a broad spectrum of biological activities and its dietary supplementation has been considered as a colon cancer chemoprotectant and for the treatment of human intestinal polyps. This paper describes a straightforward procedure for the high-yield isolation of flavonoids and tricin from aerial parts of *Huperzia brevifolia* (Hook. & Grev.) Holub, which grows on the *paramos* of Southern Ecuador. Compared to existing procedures, this method requires limited organic solvent and no chromatographic separation. In brief, the selective separation of the metabolites expressed in *H. brevifolia* was achieved by exploiting their different chemical properties under basic or acidic conditions. The identity of isolated tricin was firmly established by 1D and 2D NMR spectra. The contents of the total flavonoid fraction (TFF) and tricin in dried aerial parts of *H. brevifolia* were determined to be 4.48% *w*/*w* (44.8 mg/g) and 3.89% *w*/*w* (38.9 mg/g), respectively, using HPLC-DAD analysis. On the other hand, unoptimized crystallization of TFF delivered pure tricin in a 0.66% (*w*/*w*) yield relative to TFF, which corresponded to 2.96% *w*/*w* (29.6 mg/g) of dried aerial parts. This concentration of tricin is thus one of the highest in any plant species and makes *H. brevifolia* a valuable source of the flavone for preclinical trials and dietary supplementation for colon health.

## 1. Introduction

**Figure 1:**
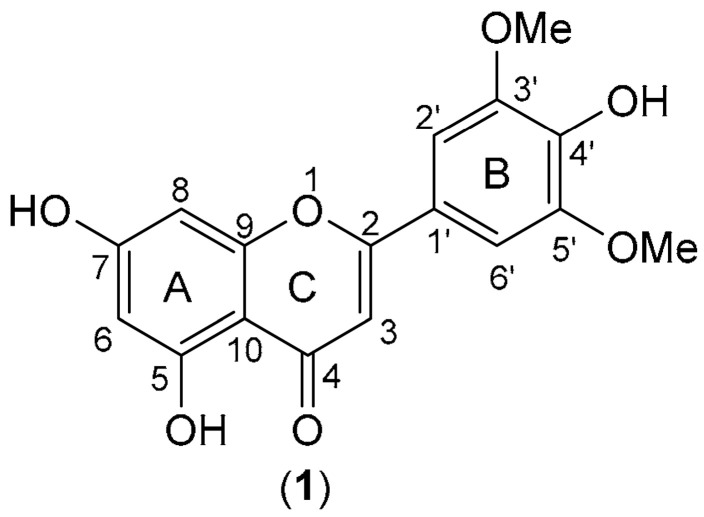
Structure of tricin (**1**).

Tricin (5,7,4′-trihydroxy-3′,5′-dimethoxyflavone; systematic IUPAC name: 5,7-dihydroxy-2-(4-hydroxy-3,5-dimethoxyphenyl)-4*H*-1-benzopyran-4-one) has the molecular formula C_17_H_14_O_7_ and structure **1** (Figure 1). It is characterized by the presence of a free OH at C-4′ and two OMe groups at C-3′ and C-5′ of a 2-phenylchromen-4-one nucleus [1]. This flavone, in its free form, is not abundant in plants compared to other flavonoids [1], though it occurs in various sources of plants and tissues [1,2]. In addition to the leaves and grains of cereal plants, such as wheat, rice, oat, maize and barley, the presence of tricin and its derivatives has been reported in bamboo, palms, sugarcane, and even in brewer’s spent grain and as pigments in insects [1,2,3].

Tricin (**1**) is one of the flavonoids with the broadest spectrum of biological activities [1]. In fact, these include antioxidant, antiradical, anti-inflammatory, antiviral, antihistaminic, anti-ulcerogenic, cancer cell antiproliferative [4] properties, and gastroprotective and antinociceptive potential [5]. Moreover, dietary tricin supplementation inhibited inflammation-related colon carcinogenesis and suppressed metastasis in colon cancer mouse models [6,7], suggesting its potential use for pre-clinical and clinical trials of colorectal cancer chemoprevention [8] and for the treatment of human intestinal polyps. Additionally, tricin showed neuroprotective and cardioprotective potential, contributing to reduced risks of cardiovascular diseases and neurodegenerative disorders [9]. In view of potential medicinal applications, extraction, isolation and quantification of tricin from natural sources have been the focus of various studies [1,2,10], as an alternative to the classical flavonoid chemical synthesis via multi-step variants of the Baker–Venkata–Raman reaction [1].

The isolated yield of free tricin varies significantly among different taxa and plant tissues, ranging from less than 1 mg to a few grams per kilogram of plant material [1,2]. The flavone is usually isolated from plants by traditional flavonoid extraction techniques, i.e., through a combination of solvent extractions, liquid–liquid partitioning, and different chromatographic separation and purification methods [1]. Advanced techniques to reduce energy and solvent consumption and increase efficiency and selectivity have recently been developed [11]; however, overall yields of tricin isolation remain low. For example, Park et al. achieved a tricin yield of 0.0329 mg/g from rice hulls using enzymatic hydrolysis combined with high hydrostatic pressure compared to 0.0147 mg/g with conventional solvent extraction [12]. Other methods, such as alkaline extraction and weak acid extraction, yielded 1.1–1.7% and 6.3% tricin from grass lignin, respectively [13].

A few years ago, we identified tricin in the aqueous methanolic extracts of a few *Huperzia* species (family Lycopodiaceae) collected in Ecuador [14]. These plants, locally known with the vernacular names of *trencillas* or *wamingas*, grow in typical neotropical Andean ecosystems called *páramos de pajonal*, at altitudes varying from 2850 to 3600 m above sea level, where the average annual temperature is 7 °C and the UV radiation is very intense. The plants are employed by visionary healers (yachak) of Saraguro communities located since the times of the Incas in the Loja province in Southern Ecuador as purgative agents and, mixed with other plants, to obtain psychotropic preparations for performing magical-ritual ceremonies and for healing supernatural diseases, such as *espanto* (startle), *susto* (fright), *mal aire* (bad air), and *shuka* (evil eye) [15]. These effects have been attributed to a large presence of alkaloids in the plants [15]. The estimated content of tricin (**1**) using the UHPLC-UV-ESIMS method in the total flavonoid fraction of aqueous methanolic extracts of *Huperzia* aerial parts varied from 0.02% (*w*/*w*) in *H. crassa* (Humb. & Bonpl. ex Willd.) Rothm. to 0.28, 0.37, 0.57 and 2.74% (*w*/*w*) in *H. kuesteri* (Nessel) B. Øllg., *H. espinosana* B. Øllg., *H. compacta* (Hook.) Trevis., and *H. brevifolia* (Hook. & Grev.) Holub, respectively [14]. A lower amount of tricin than in *H. brevifolia* was also found in *H. tetragona* (Hook. & Grev.) Rothm, *H. weberbaueri* (Nessel) Holub, and *H*. *columnaris* B. Øllg. [15].

**Figure 2:**
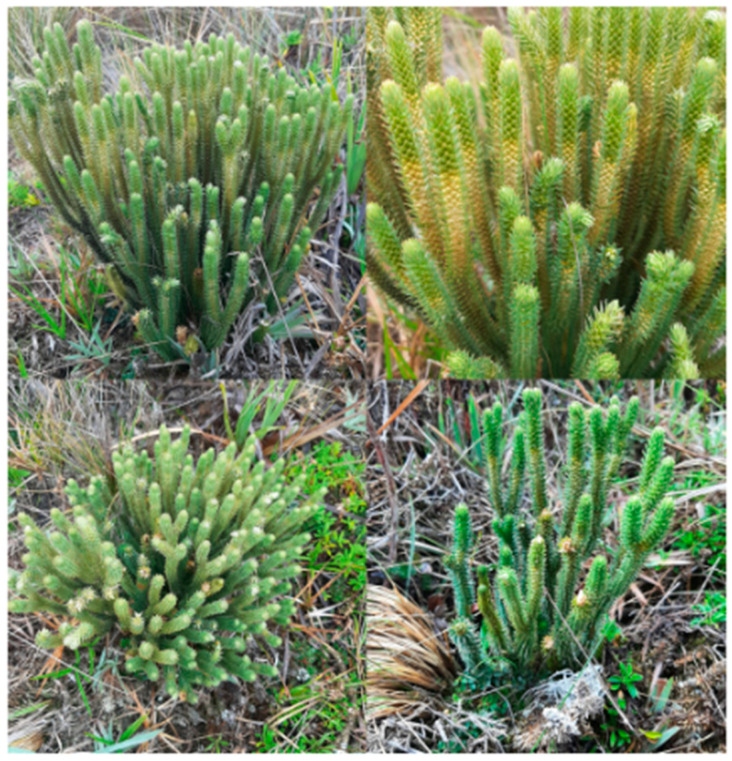
Samples of *Huperzia brevifolia* collected in Cerro Acacana, Las Antenas, Loja province (photo taken by L.N.C).

In our preliminary studies, the isolation of the flavonoid mixture required a long, tedious process that included filtration of the extract on a C-18 SPE cartridge, followed by semipreparative separations on RP-18 TLC plates [14,15]. Therefore, we decided to develop a specific, time-efficient, and cost-effective protocol for the high-yield isolation of a tricin-enriched flavonoid fraction from *H. brevifolia* (Figure 2), which could afford pure flavone (**1**) via simple crystallization. Our main aim was to avoid any chromatographic separation and reduce the use of organic solvents.

This paper describes a straightforward procedure for the extraction of flavonoids and tricin from aerial parts of *H. brevifolia*.

## 2. Results

Previous phytochemical studies have shown that alkaloids, flavonoids, and serratane triterpenoids, in varying quantities, are the most characteristic secondary metabolites extracted from *Huperzia* species [14]. The first two families are especially expressed in *H. brevifolia*, while triterpenoids are minor constituents. We hypothesized that selective separation of flavonoids from alkaloids and other metabolites could be achieved based on the different partitioning of compounds between organic solvents and aqueous solutions and on their property to form water-soluble salts with acids or bases.

**Figure 3:**
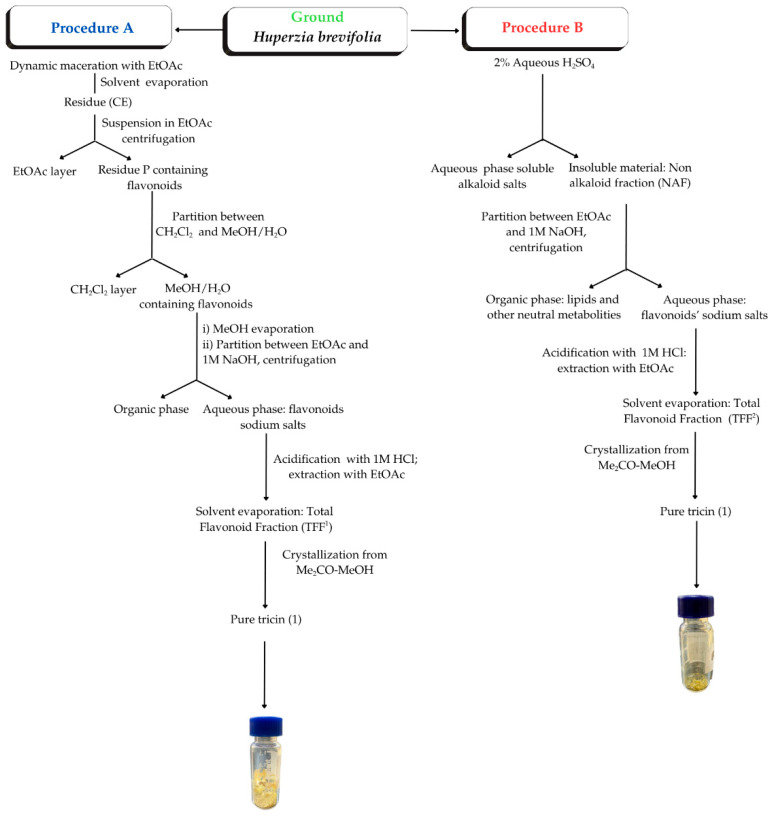
Flow chart of procedures A and B for extracting flavonoids and tricin (**1**) from *Huperzia brevifolia.*

Two procedures (A and B in Figure 3) were thus explored, using the same lot of botanical material. Procedure A was mainly based on liquid–liquid partitioning, while the different acidic and basic properties of metabolites were exploited in procedure B. However, both processes A and B finally afforded a total flavonoid fraction (TFF^1^ and TFF^2^, respectively) rich in tricin (**1**), which was readily obtained in pure form by crystallization. The content of tricin in the different fractions was monitored with HPLC-DAD analyses. Finally, the identity of purified tricin (**1**) was firmly established by ^1^H- and ^13^C-NMR spectroscopy.

In procedure B (Figure 3), flavonoids, as sodium salts, were separated from a mixture of non-alkaloid metabolites (NAF) dissolved in EtOAc. To facilitate the extraction and the separation of the two phases, the mixture was centrifuged; subsequently, free alkaloids were recovered by acidification of the salts. To evaluate the influence of the centrifugation time (CT) and the pH of the acidified solution on the yields of TFF^2^ and tricin from procedure B (see Section 4.5.2), four independent experiments were performed in triplicate using 1 g of NAF, changing the CT and the pH (entries 2–5 in Table 1). The results (mean ± standard deviation (SD)) of these experiments were compared with an analogous experiment (entry 1 in Table 1) performed in triplicate on residue P resulting from the extraction of *H. brevifolia* aerial parts with procedure A (Figure 3).

The highest yields of the total flavonoid fraction and tricin (**1**) were obtained with procedure B, using a centrifugation time of 20 min and a solution acidified to pH = 5 for the extraction of free flavonoids (entry 3 in Table 1). Under these conditions, tricin concentration in the total flavonoid fraction and in the extracted aerial parts reached the highest values of 86.84% and 3.89% *w*/*w* (38.9 mg/g), respectively, compared to 42.92% and 0.56% (5.6 mg/g), respectively, determined for procedure A (entry 1 in Table 1). On the other hand, non-optimized crystallization of the TFF^2^ from experiment 3 afforded 111.26 mg of pure tricin (**1**) in 0.66% yield, which corresponds to a yield of 2.96% *w*/*w* (29.6 mg/g) relative to ground dried aerial parts of *H. brevifolia*.

### ANOVA and Tukey Analysis

Table 2 shows the analysis of variance of the TFF^2^ yield determined for the four experiments (entries 2–5 in Table 1) performed on samples of non-alkaloid fractions (NAFs) obtained with procedure B (Figure 3), to facilitate the comparison with experiment 1 (Figure 3 and entry 1 in Table 1). The pH (df = 1) exhibited a *p*-value ≥ 0.05 that exceeded the established significance level. This result indicated that the flavonoid extraction yields were not significantly affected by pH changes between 4 and 5, possibly indicating that the solubility of flavonoids in the extraction solvent did not vary significantly with the pH. In contrast, the centrifugation time CT) markedly affected the results (df = 1, *p* ≤ 0.05), suggesting an optimal time for the efficient extraction of flavonoid salts from the matrix and the separation of the organic and the aqueous phases. Finally, the interaction between the pH and the centrifugation time was not statistically significant (df = 1, *p* ≥ 0.05). Statistical analysis indicated that the model associated with the pH, CT, and their interaction factors is normally distributed and exhibits evident homoscedasticity.

**Figure 4:**
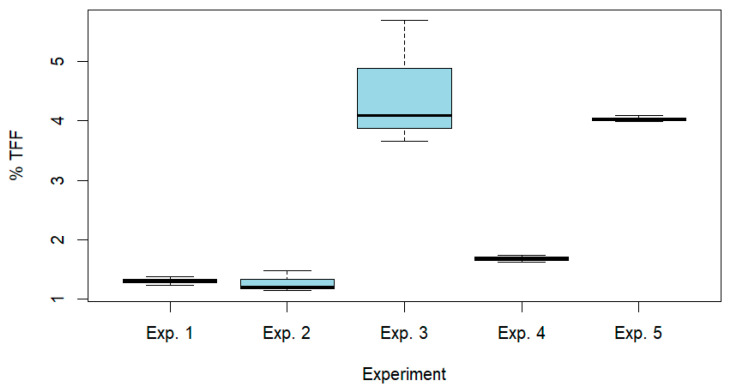
Percentage of total flavonoid fractions (TFF) in the experiments performed (details in Table 1).

Tukey’s post hoc analysis revealed that TFF and tricin yields did not vary significantly for a pH of 4 or 5 (adjusted *p* = 0.9088). In contrast, a centrifugation time of 20 or 15 min led to significantly different yields (adjusted *p* = 0.05), with the higher yields obtained with 20 min of centrifugation. On the other hand, the yields were significantly influenced by the combination of the pH and the centrifugation time, with notable differences observed between the following pH/CTpairs: 4/20 vs. 4/15; 5/20 vs. 4/15; 4/20 vs. 5/15; and 5/20 vs. 5/15. Overall, these results indicated that the centrifugation time (CT) was the primary factor determining the yields of TFF and tricin, while the pH value had a marginal effect. Figure 4 represents a boxplot with the distribution of % TFF in the five experiments 1–5 (Table 1).

## 3. Discussion

The higher efficiency of procedure B than A for extracting flavonoids and tricin from *H. brevifolia* can be attributed to its being more specific for flavonoids. Moreover, in procedure A, the extraction of flavonoids from aerial parts with EtOAc may have been incomplete, and some loss of flavonoids may have occurred due to the non-selective partitioning between solvents. On the other hand, it is known that tricin and flavonoids are often present in plants as *O*-glycosides [1,2]. Therefore, as one reviewer has suggested, the yield of flavonoid content, including tricin, may have been improved in procedure B due to some enzymatic hydrolysis [16] and cleavage of the corresponding *O*-glycosides or acetates [1] by the 2% H_2_SO_4_ used for the extraction of the botanical material. Thus, to eliminate this possibility, the contact time with the acid was limited to 5 min, and the extraction was conducted using an acid solution cooled to −10 °C. On the other hand, harsh conditions are normally needed for the hydrolysis of flavonoid glycosides [17]. For example, in a recent work, to hydrolyze tricin *O*-glycosides, they were treated with methanol–15% hydrochloric acid (4:1, *v*/*v*) in a thermostat boiling-water bath at a temperature of 85 °C for 15 min [18].

In summary, the estimated content of free tricin (**1**) in *H. brevifolia* aerial parts is significantly greater than in the top ten tricin-rich botanicals/medicinal herbs [2] and in different tissues of selected Gramineae [18,19,20,21], bamboo [22], and other species [23]. The high content of flavonoids and tricin in *H. brevifolia* may depend significantly on the climatic conditions for growth. In fact, due to the high altitude and the equatorial latitude of the *trencillas*’ characteristic habitat, that is, the *páramo de pajonal* [24], the tissues of *H. brevifolia* are exposed to strong UV rays, against which high amounts of antiradical flavonoids are produced for protection.

Regarding the yield of the total non-alkaloid fraction (NAF) (procedure B in Figure 3), it is interesting to compare the value of 26.6% relative to ground dried plant material (*w*/*w*) with the NAF values (*w*/*w*) determined for *H. kuestery, H. espinosana, H.compacta*, *H. tetragona*, *H. weberbaueri*, and *H*. *columnaris*, which ranged from 7.1% for *H. tetragona* to 12.1% for *H. kuesteri* [14,15]. This variation suggests significant differences in the composition of specialized metabolites occurring in the aforementioned *Huperzia* plants, especially as concerns the abundance of alkaloids, lipids, serratane triterpenoids and flavonoids [15]. Therefore, while *H. brevifolia* is the choice species for the extraction of TFF and tricin; other *Huperzia* species may serve as sources of the other metabolites. Moreover, although the crystallization of tricin (**1**) was not optimized in this work, procedure B, under the conditions of entry 3 in Table 1, is the method of choice for the high-yield extraction of pure tricin from *H. brevifolia*.

In addition, to evaluate the economic feasibility of the extraction methodology B developed in this work, a price of $11.16/mg was roughly estimated for tricin (**1**), upon quantifying the direct and indirect costs of the procedure. In comparison, the price of tricin, with a purity of ≥ 90%, sold by the U.S.-based company ChromaDex (Irvine, CA, USA), is $51.8/mg.

In summary, *H. brevifolia* can be considered a competitive natural source of flavone **1**, compared to other botanicals.

## 4. Materials and Methods

### 4.1. Solvents and Reagents

Except where indicated otherwise, all solvents and reagents employed in this study were purchased from Merck/Sigma-Aldrich (Saint Louis, MO, USA).

### 4.2. Instruments

1D and 2D NMR spectra were recorded in DMSO-d_6_, in a 5 mm prodigy probe with a Bruker MSC 10201 500 MHz NMR spectrometer (Billerica, MA, USA) using the TopSpin 4.5.0. spectrometer software. HPLC Analyses were performed using a HPLC-DAD instrument (Dionex UltiMate 3000, Thermo Fisher Scientific, Waltham, MA, USA), equipped with a reversed-phase C18 column, 250 mm × 4.6 mm, 5 µm (Thermo Fisher Scientific, Waltham, MA, USA). The melting point of tricin was determined with a Fisher-Johns melting point apparatus (Fisher Scientific Italia, 20054 Segrate (MI), Italy).

### 4.3. Botanical Material

Aerial parts of *H. brevifolia* were collected on the *páramo* grasslands (sandy-loam soil type with black color and high humidity) of Cerro Acacana, Las Antenas, located at the border between Saraguro and San Lucas parish (3°37′31″ S; 79°14′28″ W), during May 2023, a month that is characterized by being the rainy season with 10 °C, and rainfall of 80 mm on average, respectively, in the province of Loja in southern Ecuador. The species was identified by Bolivar Merino, curator of the “Herbarium Reinaldo Espinoza” in the Universidad Nacional de Loja (HUNL); a voucher specimen has been deposited in the Herbarium of the Universidad Técnica Particular de Loja (UTPL) with the accession code PPNIc-10.

### 4.4. Pre-Treatment of Plant Material

Aerial parts were dried in the shade at 22 °C for 3 days under a constant flow of dry air. Subsequently, they were ground to a size of 40 µm using an Ultracentrifugal Mill ZM 200 (Fisher Scientific, Beijing, China), and the resulting powder was stored in glass containers at room temperature until use.

### 4.5. Extraction of Flavonoids and Tricin from H. brevifolia

#### 4.5.1. Procedure A: Extraction of Plant Material with EtOAc

Finely ground plant material (5 g) was subjected to dynamic maceration in EtOAc (1:50 *w*/*v*) for 8 h at 22 °C. The extract was filtered on a sintered glass filter and evaporated under vacuum with a rotary evaporator [Hei-VAP Value-hand lift G1 standard model (Fisher Scientific, Beijing, China)] in a water bath heated to 40 °C. Subsequently, the resulting residue (CE, 0.44 g, 8.8% of dried plant material (*w*/*w*)) was resuspended in EtOAc (40 mL) and the mixture was centrifuged at 22 °C for 15 min, with a Sorvall^TM^ ST 8 centrifuge (Thermo Fisher Scientific, Waltham, MA, USA) at 12,000 RFC. An insoluble fraction (P) containing flavonoids (UV detection on TLC plates, NH_3_ fumes) [25] was separated. Subsequently, residue P was partitioned between CH_2_Cl_2_ (15 mL) and MeOH-H_2_O, 1:1 (2 × 15 mL), followed by distilled water (3 × 10 mL). The combined MeOH and H_2_O fractions, containing flavonoids including tricin (**1**), were concentrated at 40 °C to eliminate MeOH. The remaining aqueous layer was treated with cold (0 °C) 1 M NaOH (15 mL), and then EtOAc (30 mL) was added. The two phases were centrifuged at 12,000 RFC at 0 °C °C for 15 min with an Eppendorf refrigerated centrifuge model 5427 R (Eppendorf S.r.l., Milan, Italy) and separated; subsequently, the alkaline fraction containing flavonoid salts was acidified to pH 5 with cooled to −10 °C 1 M HCl. Finally, the aqueous acidic solution was extracted quickly with EtOAc (3 × 50 mL). Solvent evaporation under vacuum at 40 °C afforded 65.6 mg of the total flavonoid fraction (TFF^1^, 1.31 ± 0.18% of extracted plant material, 13.12 mg/g; estimated (HPLC-DAD) tricin content = 28.16 mg (42.92 ± 1.95% of TFF^1^ and 0.56% (*w*/*w*) (5.63 mg/g) of extracted plant material) (entry 1 in Table 1). Subsequently, TFF^1^ was exposed to Me_2_CO (2 mL) in which it was only partially soluble. After removal of the supernatant, the acetone-insoluble fraction (20 mg) was dissolved in MeOH (1 mL). The methanolic solution was stored in a vial at 4 °C for ten days until tricin (**1**) (18 mg, 0.36% (*w*/*w*) (3.6 mg/g) of extracted plant material) crystallized. The pale-yellow crystals, mp 288–290 °C, were collected and analyzed by NMR spectroscopy, which confirmed the identity of tricin.

#### 4.5.2. Procedure B: Direct Treatment of Plant Material with Aqueous H_2_SO_4_

Finely ground aerial parts of *H. brevifolia* (200 g) were directly treated with 2% aqueous H_2_SO_4_ (3 × 80 mL), cooled to −10 °C, for 5 min each time. The resulting suspension was filtered under vacuum through a Buchner funnel, and the insoluble, alkaloid-free (negative Dragendorff test for alkaloids [26,27]) material [Non-Alkaloid Fraction (NAF); 53.2 g, 26.6% of extracted plant material] was wetted with cold 10% aqueous NH_3_ to neutrality. The acidic aqueous solution, containing alkaloids as sulfate salts, was extracted with hexanes (3 × 50 mL) to remove most lipids and chlorophylls. Subsequently, it was treated with aqueous NH_3_ to pH 12 to release alkaloids as free bases, which were extracted with CHCl_3_ until a negative response to Dragendorff reagent [26,27]. The chloroform solution was then dried (Na_2_SO_4_), concentrated at 22 °C under vacuum and the alkaloid-rich residue (AF) was stored in an amber vial for a separate investigation. The NAF was used for the isolation of flavonoids. To this purpose, a NAF sample (1 g) was partitioned between EtOAc (25 mL) and cold (0 °C) 1 M aqueous NaOH (3 × 15 mL). The two phases were centrifuged with a refrigerated centrifuge at 12,000 RFC; subsequently, the separated alkaline fraction containing flavonoids as sodium salts was acidified with cold (−10 °C) 1 M aqueous HCl. Finally, the released free flavonoids were extracted with EtOAc (4 × 25 mL). Solvent evaporation gave TFF^2^, which afforded pure tricin (**1**) by crystallization from Me_2_CO-MeOH, following the procedure described in Section 4.5.1.

### 4.6. Procedure B: Variation in the Centrifugation Time and the pH of the Acidified Solution

Four experiments were performed in triplicate on four NAF samples (1 g each) to evaluate the effects of the pH of the acidified solution of flavonoids and the centrifugation time on the yields of TFF^2^ and tricin. The procedure described in Section 4.5.2 was followed in all experiments. The tricin content in TFF^2^ was determined for each experiment by HPLC-DAD (see Section 4.7) and compared with experiment 1 (see Section 4.5.1). The data, reported in entries 2–5 in Table 1, are represented as the mean ± SD, and were analyzed, as well as their interaction, using an ANOVA model. Subsequently, Tukey’s test was applied to identify specific differences between the experiments. The statistical analysis was performed using R-studio software 4.0 [28].

### 4.7. Tricin Quantification by HPLC-DAD

The methodology used for tricin quantification was based on procedures previously reported in the literature [29,30], with minor modifications. Detailed chromatographic conditions employed for tricin quantification by HPLC-DAD are presented in Table 3. Before analysis, each sample was filtered through Titan3™ PTFE (Hydrophilic) Syringe Filters, 0.2 μm, 17 mm (42213-NPL, Thermo Fisher Scientific, Waltham, MA, USA). Analyses were performed using the following chromatographic conditions: isocratic elution with a mobile phase consisting of 60% MeCN (purity ≥ 99.9%) and 40% deionized H_2_O; eluent flow rate = 1 mL/min; volume of each injected solution (1 mg sample/mL MeOH) = 5 μL; auto-sampler compartment and column temperature set at 30 °C and 50 °C, respectively; total run time = 15 min. The UV spectra were recorded from 200 to 400 nm, whereas tricin was monitored at 254 nm, Tricin, eluted with a retention time of 3.4 min (Figure S1 in the Supplementary Materials), was quantified by integrating the area under the peak and interpolation from a calibration curve generated from a 500 ppm stock solution of standard tricin (Sigma Aldrich, Saint Louis, MO, USA) in MeOH. The solution was diluted with MeOH to eight concentrations (0.75, 1.5, 3, 6, 12, 25, 50, 100 ppm), and each solution was analyzed 3 times by HPLC-DAD to determine the accuracy and repeatability of the method (inter-assay precision), using the same conditions as the sample. The limit of detection (LD, S/N = 3) and limit of quantification (LQ, S/N = 10) of tricin were 6.853 ppm and 57.129 ppm, respectively, with the correlation coefficient R^2^ = 0.9998. In the considered concentration range, the method showed how its linearity and sensitivity corresponded to the slope of the calibration line y = 3386.51x − 18,842. The relative standard deviations for the inter-day and intra-day assays were <5% The values of % tricin in the TFF reported in Table 1 are the average of 3 measurements.

### 4.8. Tricin Identification

The identity of tricin (**1**), yellow crystals, mp 288–290 °C from MeOH, was firmly confirmed by 1D and 2D NMR spectra (Figures S2–S8 in the Supplementary Materials) that were recorded in DMSO-d_6_ in a 5 mm prodigy probe with a Bruker MSC 10201 500 MHz NMR spectrometer (Billerica, MA, USA) using the TopSpin 4.5.0. spectrometer software. Chemical shifts are reported in δ (ppm); coupling constants (*J*) are in Hz. The multiplicity of carbon signals was determined by DEPT experiments.

^1^H NMR (500 MHz, DMSO-d_6_): δ_H_ 3.87 (2 × equivalents CH_3_, two coincident s, 3′-OMe and 5′-OMe), 6.17 (1H, d, *J* = 2, H-6), 6.51 (1H, d, *J* = 2, H-8), 6.87 (1H, s, H-3), 7.26 (2 × equivalent Hs, two overlapped s, H-2′ and H-6′), 9.35 (s, 1H, 4-OH), 10.81 (1H, s, 7-OH), 12.95 (1H, s, 5-OH).

^13^C NMR (125 MHz, DMSO-d_6_): δ_C_ 56.8 (two equivalent carbons, 2× CH_3_, 3′ and 5′-OCH_3_), 94.7 (CH, C-8), 99.3 (CH, C-6), 104.0 (CH, C-3), 104.2 (C, C-10). 104.9 (two equivalent carbons, 2 × CH, C-2′ and C-6′), 120.9 (C, C-1′), 140.4 (C, C-4′), 148.7 (two equivalent carbons, 2 × C, C-3′ and C-5′), 157.8 (C, C-9), 161.9 (C, C-5), 164.1 and 164.6 (2 × C, C-2 and C-7), 182.2 (C, C-4).

The NMR data corresponded well to the literature [2,17,23].

## 5. Conclusions

In the simple, time-efficient, and cost-effective protocol (procedure B in Figure 3) developed in this work, the total flavonoid fraction, including the bioactive flavone tricin (**1**), was extracted from *H. brevifolia* using a limited amount of organic solvent and no tedious chromatographic separation. These findings may have important implications for natural product chemistry and isolation strategies. The estimated outstanding content (3.89%, 38.9 mg/g) of tricin (**1**) in *H. brevifolia* dry aerial parts is higher than that for other known botanical organisms and underscores the potential of *H. brevifolia* as a valuable natural source of tricin *,* which has growing pharmaceutical and nutraceutical interest [4,31,32]. However, our findings are based on a single instance of plant collection; therefore, future research should explore the reproducibility of tricin content across different populations of *H. brevifolia* and conditions.

## Figures and Tables

**Table 1:** Estimated amounts of the total flavonoid fraction (TFF) and tricin (**1**) ^±^ in aerial parts of *Huperzia brevifolia.*

Experiment	CT (min)	pH	TFF^1^	TFF^2^	Tricin Content
1	15	5	1.31 ± 0.18 * 13.1 ± 1.8 **		42.92 ± 1.95 ^†^ (0.56) ^§^ (5.6) ^§§^
2	15	5		1.31 ± 0.07 * 13.1 ± 0.7 **	78.37 ± 0.40 ^†^ (1.02) ^§^ (10.2) ^§§^
3	20	5		4.48 ± 1.07 * 44.8 ± 10.7 **	86.84 ± 0.37 ^†^ (3.89) ^§ ^ (38.9) ^§§^
4	15	4		1.68 ± 0.06 * 16.8 ± 0.6 **	21.10 ± 1.27 ^†^ (0.35) ^§^ (3.5) ^§§^
5	20	4		4.03 ± 0.05 * 40.3 ± 0.5 **	46.69 ± 0.22 ^† ^(1.88) ^§^ (18.8) ^§§^

^±^ Quantified by HPLC-DAD; * percentage of total flavonoid fraction (TFF) in ground dry aerial parts *(w*/*w)*; ** mg of TFF/g dry aerial parts; ^†^ percentage of tricin in TFF *(w*/*w)*; ^§^ percentage of tricin in ground dry aerial parts *(w*/*w)*; ^§§^ mg of tricin/g dry aerial parts.

**Table 2:** Data of ANOVA analysis.

Variable ^§^	df	ss	ms	F-Value	*p*-Value	Significance
pH	1	0.001	0.001	0.009	0.925	
CT	1	22.853	22.853	403.998	3.92 × 10^−8^	***
pH:CT	1	0.056	0.056	0.991	0.349	
Residuals	8	0.453	0.057			

^§^ df = degrees of freedom; ss = sum of squares; ms = mean square. Differences are considered significant at *p* ≤ 0.05. Significance codes: *** (*p* < 0.001).

**Table 3:** Chromatographic conditions for the quantification of tricin by HPLC-DAD.

Parameter	Value
Working mode	Isocratic
Mobile phase	MeCN:H_2_O (60:40, *v*/*v*)
Flow rate (mL/min)	1.0
Column	Reversed-phase BDS HYPERSIL C18 (250 mm × 4.6 mm, 5 µm)
Column temperature (°C)	50
Detection wavelength (nm)	254
Injection volume (µL)	5
Total run time (min)	15
Retention time of tricin (min)	3.4
Sample concentration (mg/mL)	1.0

## Data Availability

The original contributions presented in this study are included in the article/Supplementary Material. Further inquiries can be directed to the corresponding author.

## Supplementary Materials

The following supporting information can be downloaded at https://www.mdpi.com/article/10.3390/plants14203220/s1. Figure S1: HPLC-DAD chromatogram of the total flavonoid fraction obtained in experiment 3 (Table 1) showing the peak of tricin (**1**); Figures S2 and S3: ^1^H- and ^13^C-NMR spectra of tricin; Figures S4 and S5: ^13^C-DEPT and ^1^H–^1^H COSY spectra of tricin; Figures S6–S8: HMBC, HMQC, and HSQC spectra of tricin (**1**).
