# Supplementary material for: A Straightforward Procedure for the High-Yield Extraction of Tricin and Flavonoids from Ecuadorian Huperzia brevifolia (Lycopodiaceae)
Source: Plants (Basel). 2025 Oct 20;14(20):3220. doi: 10.3390/plants14203220 (PMC12567224; doi:10.3390/plants14203220)
Supplement: Supplementary file 1 [file plants-14-03220-s001.zip › plants-3846475-supplementary.pdf]

plants

SUPPLEMENTARY MATERIAL

A straightforward procedure for the high-yield extraction of tricin and flavonoids from Ecuadorian *Huperzia brevifolia* (Lycopodiaceae)

Chabaco Armijos [1]*, Leydy Nathaly Castillo [1], Jorge Ramírez[1], and Giovanni Vidari [2]

[1] Departamento de Química, Facultad de Ciencias Exactas y Naturales, Universidad Técnica Particular de Loja, Loja 1101608, Ecuador
[2] Department of Medical Analysis, Faculty of Applied Science, Tishk International University, Erbil 44001, Iraq
* Correspondence: cparmijos@utpl.edu.ec

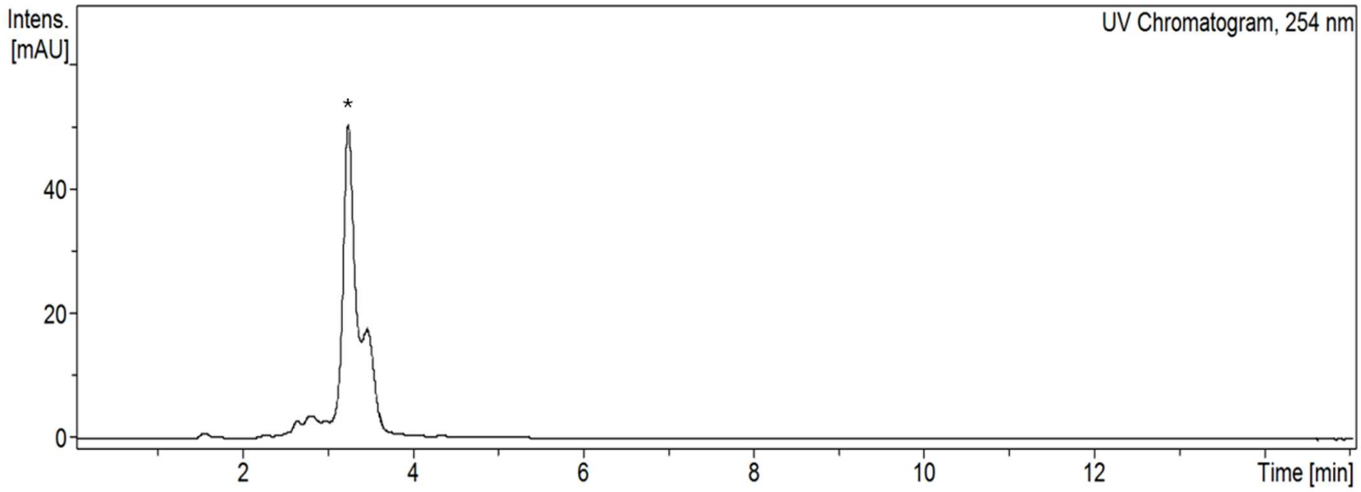

Figure S1. HPLC chromatogram of TFF[2] from experiment 3 in Table 1 showing the peak* of tricin (**1**).

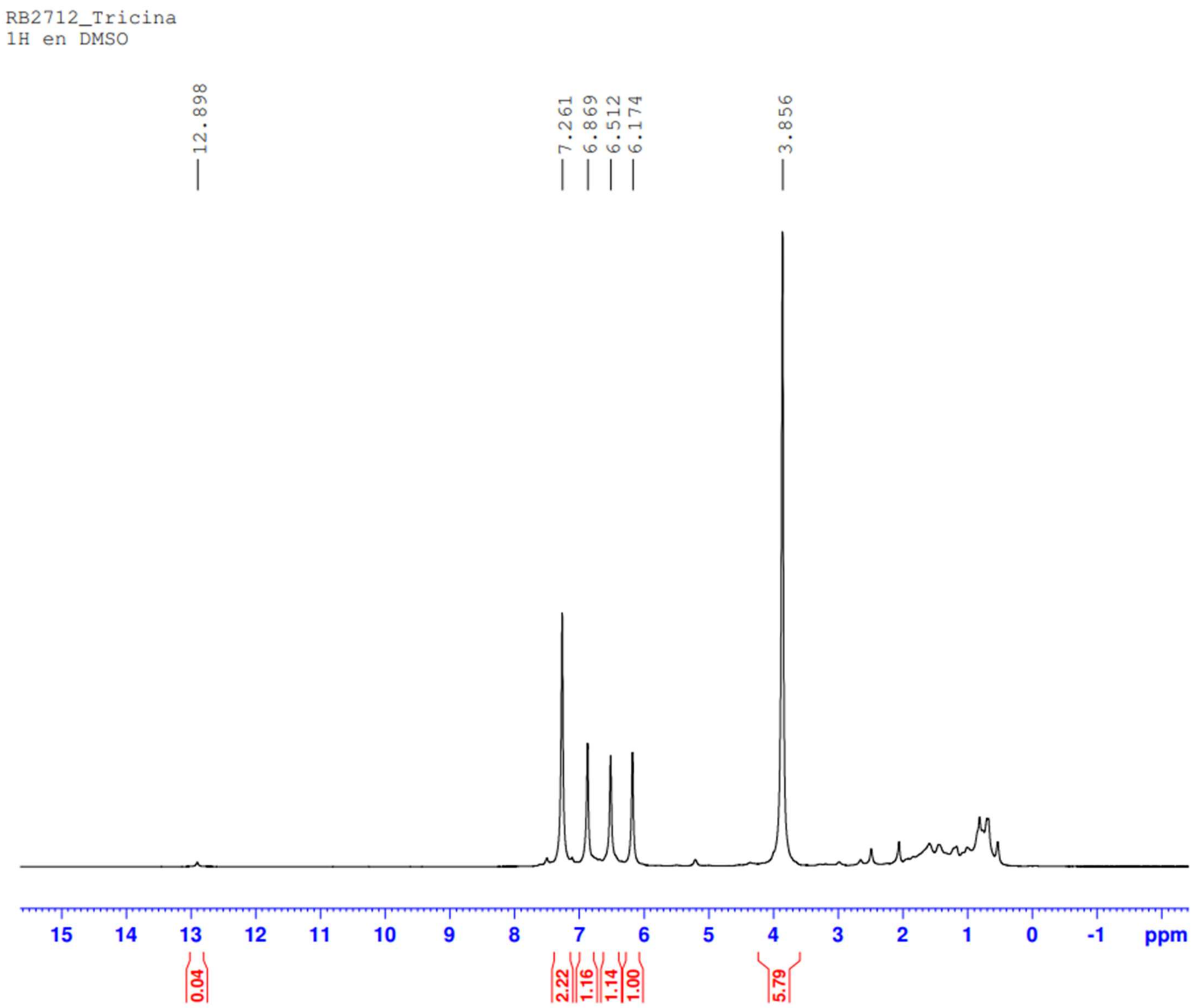

Figure S2. ^{1}H NMR (500 MHz, DMSO-d_6) spectrum of tricin (**1**).

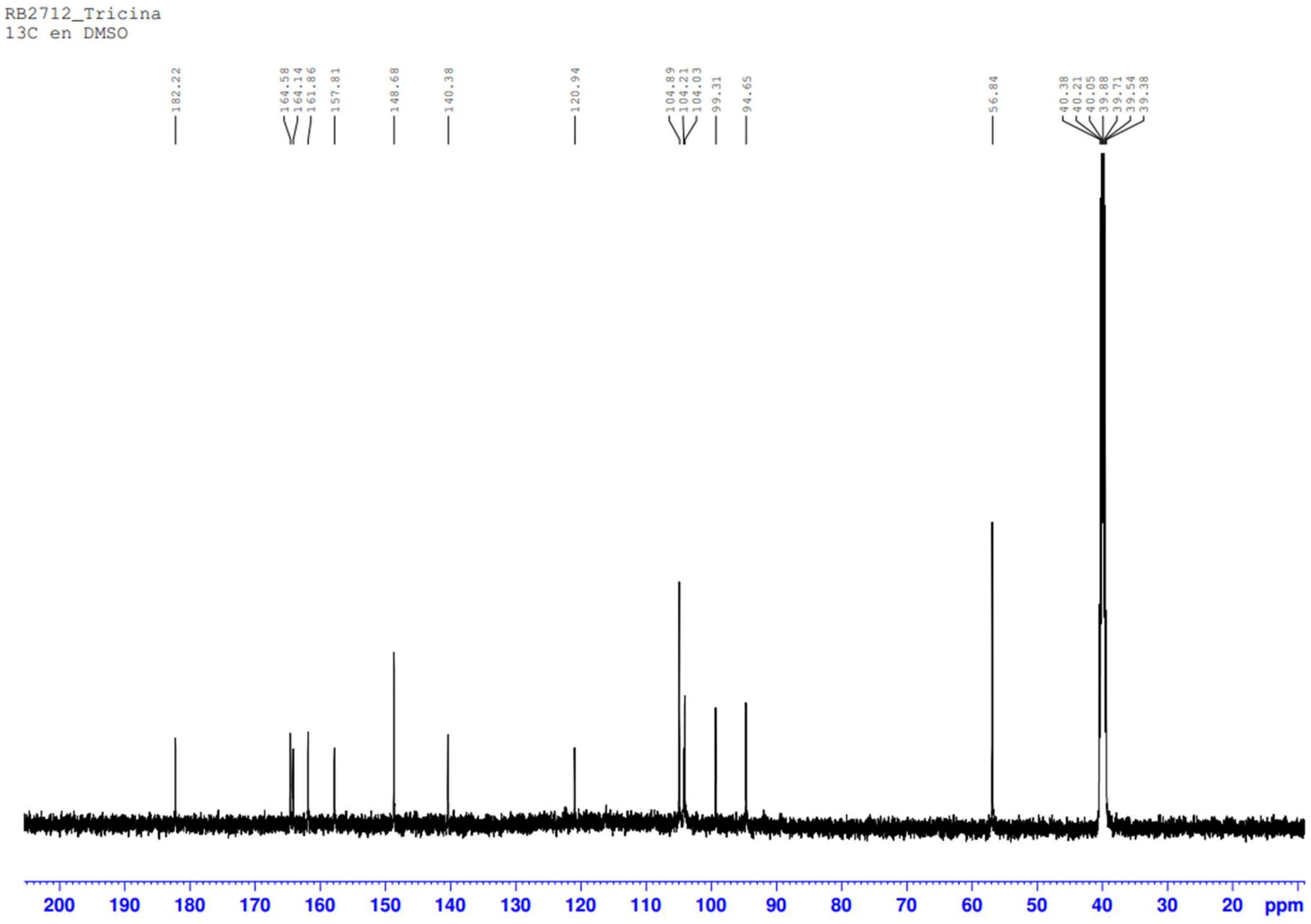

Figure S3. ^{13}C NMR (125 MHz, DMSO-d_6) spectrum of tricin (**1**).

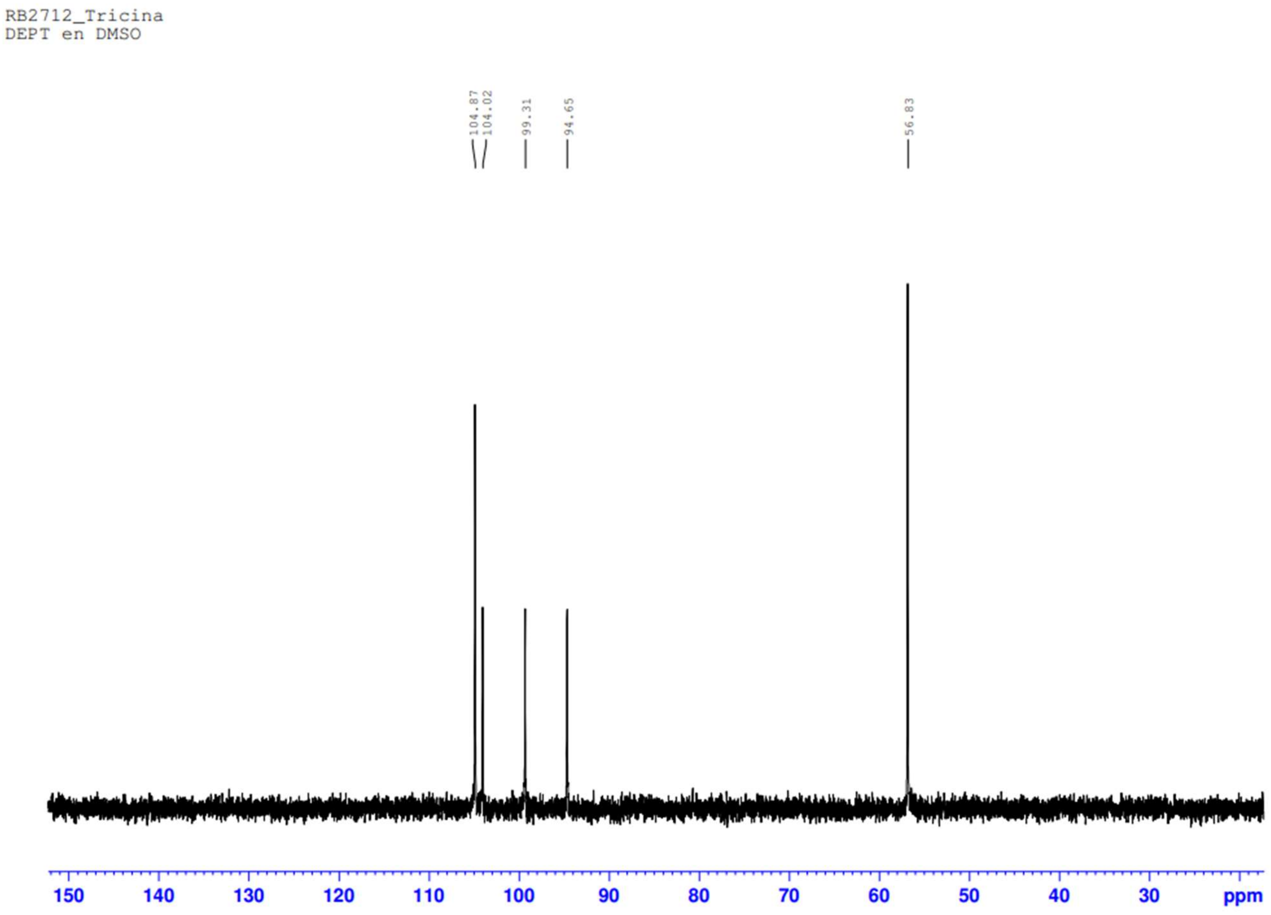

Figure S4. ^{13}C-DEPT NMR (125 MHz, DMSO-d$_6$) spectrum of tricin (**1**).

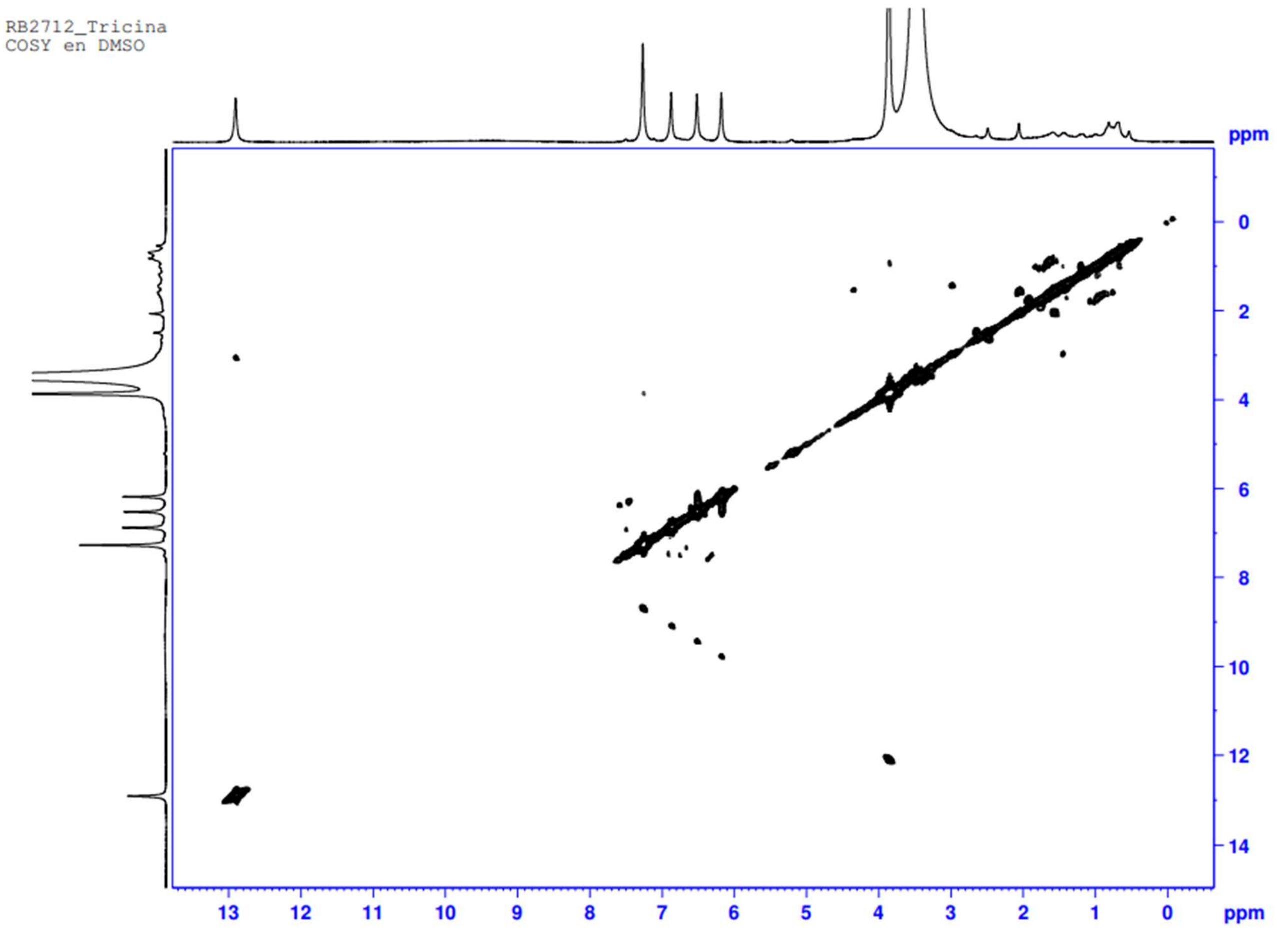

Figure S5. COSY-NMR (500 MHz, DMSO-d_6) spectrum of tricin (**1**).

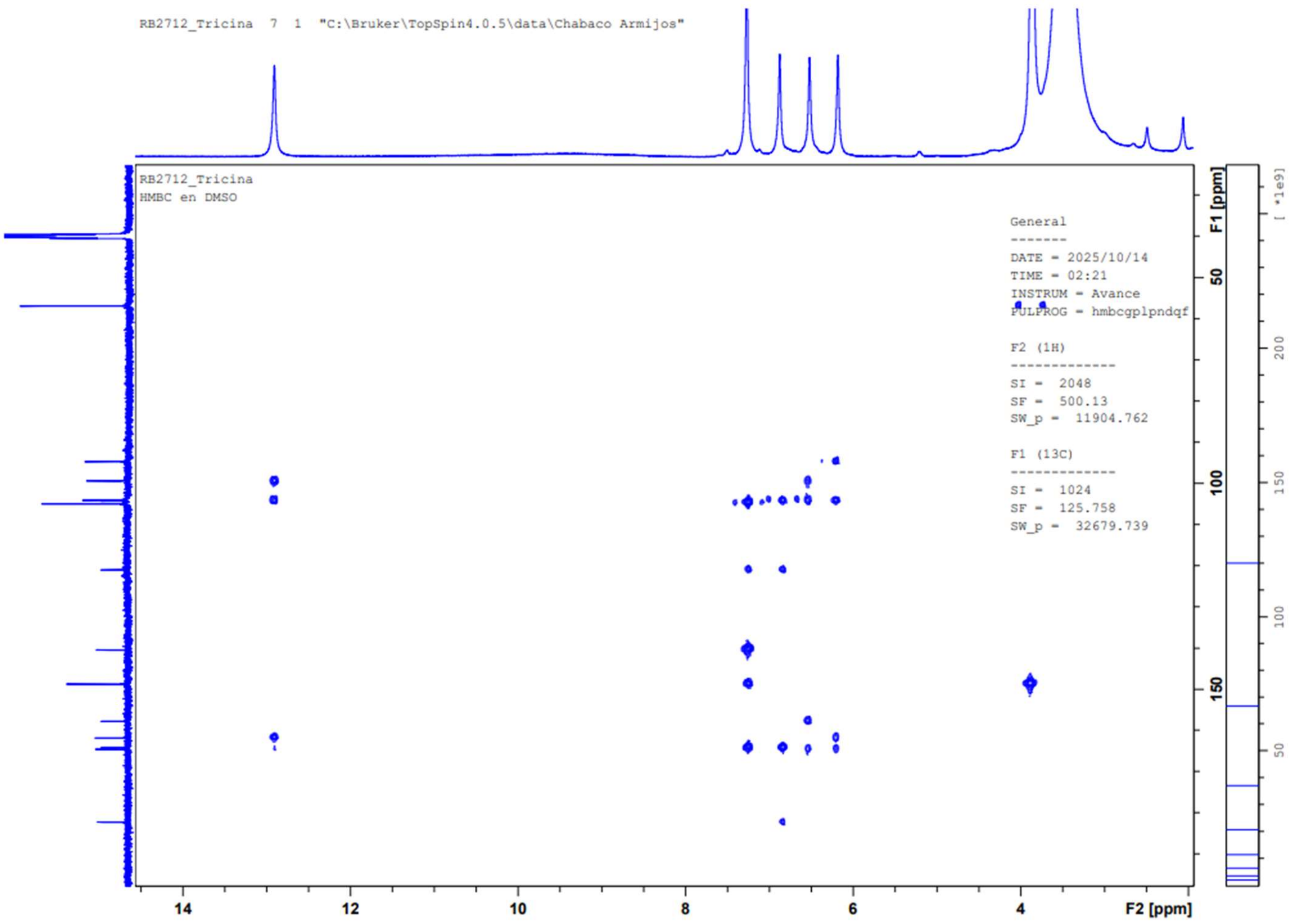

Figure S6. ^{1}H-^{13}C HMBC NMR (DMSO-d$_6$) spectrum of tricin (**1**).

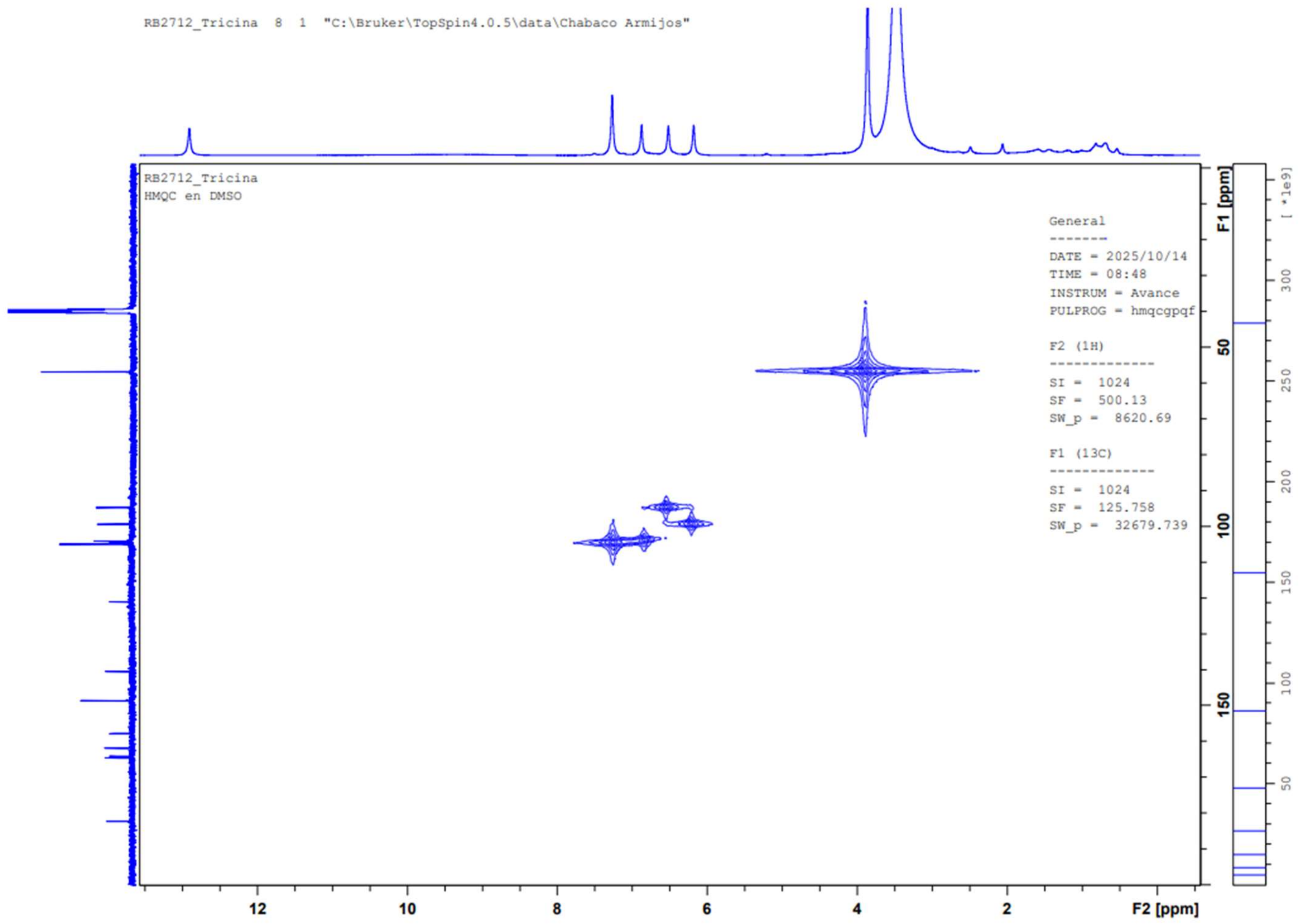

Figure S7. ^{1}H-^{13}C HMQC NMR (DMSO-d$_6$) spectrum of tricin (**1**).

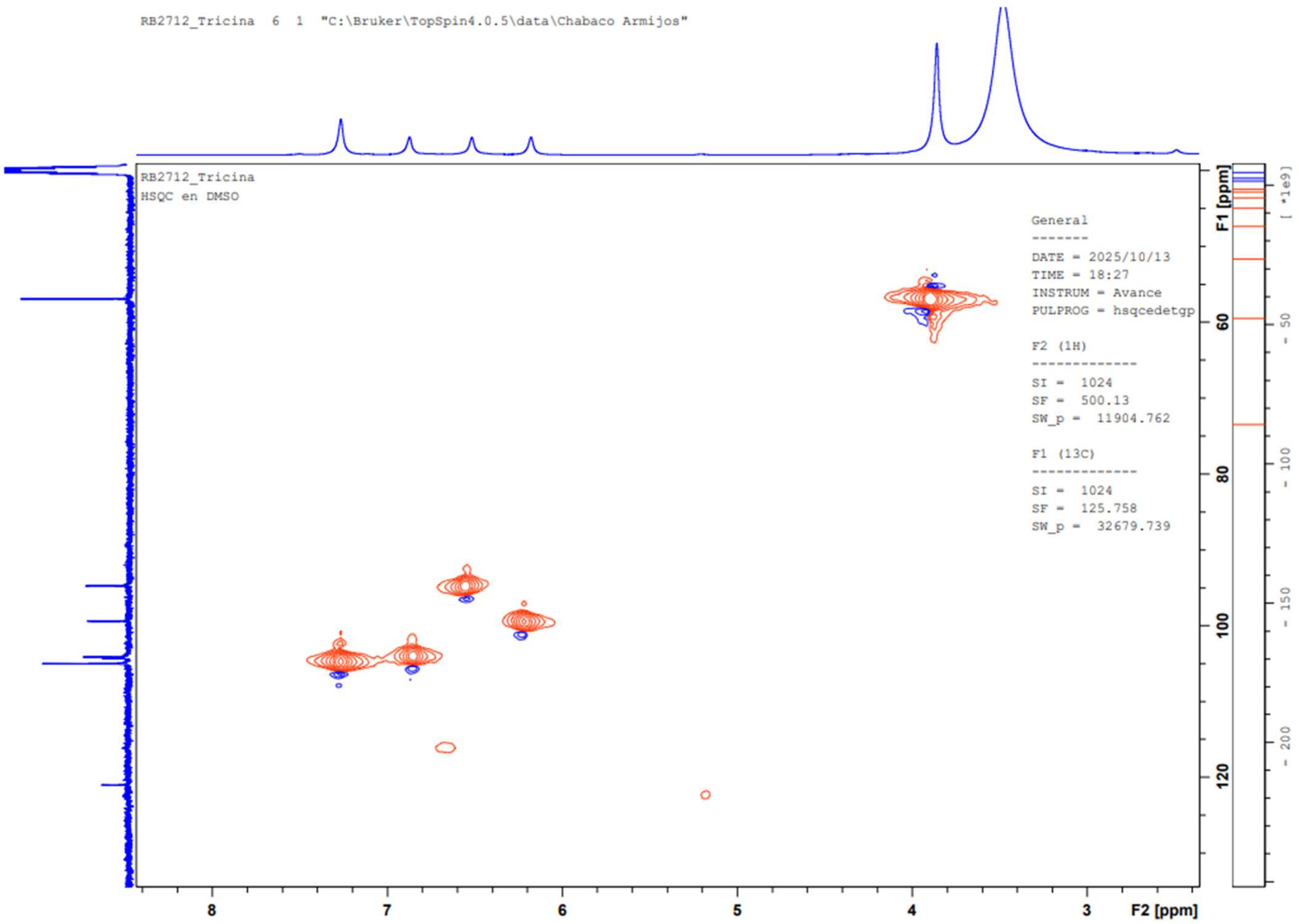

Figure S8. ^{1}H-^{13}C HSQC NMR (DMSO-d_6) spectrum of tricin (**1**).
